# Applying machine learning classifiers to automate quality assessment of paediatric dynamic susceptibility contrast (DSC-) MRI data

**DOI:** 10.1259/bjr.20201465

**Published:** 2023-02-20

**Authors:** Stephen J. Powell, Stephanie B. Withey, Yu Sun, James T. Grist, Jan Novak, Lesley MacPherson, Laurence Abernethy, Barry Pizer, Richard Grundy, Paul S. Morgan, Tim Jaspan, Simon Bailey, Dipayan Mitra, Dorothee P. Auer, Shivaram Avula, Theodoros N. Arvanitis, Andrew Peet

**Affiliations:** 1 Physical Sciences for Health CDT, University of Birmingham, Birmingham, United Kingdom; 2 Institute of Cancer and Genomic Sciences, University of Birmingham, Birmingham, United Kingdom; 3 Department of Oncology, Birmingham Children’s Hospital, Birmingham, United Kingdom; 4 RRPPS, University Hospitals Birmingham NHS Foundation Trust, Birmingham, United Kingdom; 5 School of Biological Sciences and Medical Engineering, Southeast University, Nanjing, China; 6 Department of Psychology, Aston Brain Centre, School of Life and Health Sciences, Aston University, Birmingham, United Kingdom; 7 Radiology, Birmingham Children’s Hospital, Birmingham, United Kingdom; 8 Radiology, Alder Hey Children’s NHS Foundation Trust, Liverpool, United Kingdom; 9 Oncology, Alder Hey Children’s NHS Foundation Trust, Liverpool, United Kingdom; 10 The Children’s Brain Tumour Research Centre, University of Nottingham, Nottingham, United Kingdom; 11 Medical Physics, Nottingham University Hospitals, Nottingham, United Kingdom; 12 NIHR Nottingham Biomedical Research Centre, Nottingham, United Kingdom; 13 Radiology, Nottingham University Hospitals, Nottingham, United Kingdom; 14 Sir James Spence Institute of Child Health, Royal Victoria Infirmary, Newcastle upon Tyne, United Kingdom; 15 Neuroradiology, The Newcastle upon Tyne Hospitals NHS Foundation Trust, Newcastle upon Tyne, United Kingdom; 16 Sir Peter Mansfield Imaging Centre, University of Nottingham, Nottingham, United Kingdom; 17 Institute of Digital Healthcare, WMG, University of Warwick, Coventry, United Kingdom

## Abstract

**Objective:**

Investigate the performance of qualitative review (QR) for assessing dynamic susceptibility contrast (DSC-) MRI data quality in paediatric normal brain and develop an automated alternative to QR.

**Methods:**

1027 signal–time courses were assessed by Reviewer 1 using QR. 243 were additionally assessed by Reviewer 2 and % disagreements and Cohen’s κ (κ) were calculated. The signal drop-to-noise ratio (SDNR), root mean square error (RMSE), full width half maximum (FWHM) and percentage signal recovery (PSR) were calculated for the 1027 signal–time courses. Data quality thresholds for each measure were determined using QR results. The measures and QR results trained machine learning classifiers. Sensitivity, specificity, precision, classification error and area under the curve from a receiver operating characteristic curve were calculated for each threshold and classifier.

**Results:**

Comparing reviewers gave 7% disagreements and κ = 0.83. Data quality thresholds of: 7.6 for SDNR; 0.019 for RMSE; 3 s and 19 s for FWHM; and 42.9 and 130.4% for PSR were produced. SDNR gave the best sensitivity, specificity, precision, classification error and area under the curve values of 0.86, 0.86, 0.93, 14.2% and 0.83. Random forest was the best machine learning classifier, giving sensitivity, specificity, precision, classification error and area under the curve of 0.94, 0.83, 0.93, 9.3% and 0.89.

**Conclusion:**

The reviewers showed good agreement. Machine learning classifiers trained on signal–time course measures and QR can assess quality. Combining multiple measures reduces misclassification.

**Advances in knowledge:**

A new automated quality control method was developed, which trained machine learning classifiers using QR results.

## Introduction

Dynamic susceptibility contrast (DSC-) MRI provides estimates of perfusion in the brain,^1^ by imaging the passage of a gadolinium-based contrast agent using a dynamic T_2_ or T_2_^*^ weighted imaging sequence.^2^ The contrast agent causes local changes in T_2_ and T_2_^*^, which dynamically alter the MR signal intensity.^3^ Analysis of the resulting signal–time courses, associated with each pixel, can produce estimates of cerebral blood volume (CBV), cerebral blood flow (CBF) and vascular mean transit time (MTT).^4^ As well as DSC-MRI, perfusion can also be measured using MRI with dynamic contrast-enhanced (DCE-) MRI, arterial spin labelling (ASL) and intravoxel incoherent motion (IVIM). However, DSC-MRI offers better signal-to-noise ratio (SNR) and contrast-to-noise ratio, and a faster acquisition time.^5^

Measurement of perfusion can be used to indicate health in a range of diseases. In paediatrics, it is used to assess brain tumours, which are the leading cause of cancer-related mortality in children,^6^ as well as diseases which affect neurovasculature. Most paediatric brain tumour patients have a gadolinium injection to allow for post-contrast *T*_1_ weighted imaging so the DSC-MRI acquisition can be carried out during this injection providing information that would otherwise not be available.^7^ CBV and CBF values from DSC-MRI acquisitions in paediatric patients have been used to predict long-term survival^8^ and have been shown to correlate with tumour grade.^9,10^ These applications require accurate CBV and CBF values, therefore it is important to ensure that the DSC-MRI signal–time courses they are estimated from are of good quality.^11^

DSC-MRI is prone to motion and susceptibility artefacts, which degrade the quality of acquired data.^11^ The scanner and acquisition protocol for DSC-MRI also commonly varies from centre-to-centre, which affects the signal–time courses produced^12,13^ and the SNR of the CBV maps,^14^ limiting the clinical applicability of the technique.^15^ For example, the field strength of the scanner and acquisition parameters such as repetition time (TR), echo time (TE), voxel volume and flip angle may vary between centres. These factors affect the SNR of the acquired data, whilst TR dictates the temporal resolution.^16^ In brain tumour patients, breakdown of the blood–brain barrier (BBB) can lead to contrast agent extravasation, where the contrast agent leaks into the extravascular extracellular space (EES).^17^ Contrast extravasation can lead to *T*_1_ weighted contamination of the signal–time courses and underestimation of the CBV values, or T_2_^*^ weighted effects leading to overestimation of CBV values.^17^ These contamination effects can be reduced either by administering a pre-bolus of contrast agent,^18^ or using a low flip angle during the acquisition, to reduce the *T*_1_ weighting of the DSC-MRI sequence.^11^ Recent research has shown that the application of leakage correction is essential when using a low flip angle, single-bolus protocol.^7,19,20^

Currently, the ASFNR recommendation for quality control (QC) of DSC-MRI data is to assess signal–time courses by eye, using qualitative review (QR). This involves assessing signal–time courses for the presence of artefacts, including magnetic susceptibility (the response of a material to a magnetic field, which can result in signal loss^21^ and motion); for appropriate signal drop indicating the quality of bolus administration; and for noise spikes in the signal–time curve, suggesting that any such time points should be removed.^11^ There can be discordance between reviewers and one DSC data set contains thousands of signal–time courses, so it is not practical to assess the quality of all signal–time courses manually. In practice this means that a subset of the signal–time courses is used to assess the quality of a whole data set. An automated process based on QR, which could be applied to assess signal–time course quality on a voxel-wise basis, which could be used to provide an assessment of the overall quality of a data set, is desirable.

Previous work has shown that it is possible to define statistical thresholds and apply these to quantitative measures calculated from DSC-MRI signal–time courses to assess data quality.^22^ Machine learning (ML) classification can be used to train models to make predictions based on features extracted from a data set.^23^ This has plenty of applications in medical imaging. For example, it has been used in the pneumonia detection,^24,25^ detection and classification of Covid-19,^26^ diagnosing colorectal cancer,^27^ and assisting in planning rehabilitation care for stroke patients.^28^ ML has also been applied to DSC-MRI data for several applications, but so far it has not been used for assessing data quality. For example, it has been used in place of standard analysis techniques for DSC-MRI to provide a quicker and more robust method to estimate CBF values from raw signal–time course data,^29^ predict survival in glioma patients,^30^ and classify tumour type.^31–36^ Therefore, ML could be applied to features extracted from DSC-MRI signal–time courses to determine data quality. Any new method for assessing data quality should be established in normal brain before it is applied to diseased tissues. Undertaking such a study is a challenge in children due to ethical constraints but an appropriate alternative is to uses paediatric patients undergoing DSC-MRI scans for brain tumours, and selecting signal–time courses from slices of brain which do not contain tumour.

The objectives of this paediatric study are: to assess the discordance in QR between two reviewers, to use this QR to determine thresholds of quantitative measures of data quality and to investigate whether QR and quantitative thresholds could be used to develop an automated QC process for assessing overall data quality of a paediatric data set.

## Methods

### Patient data

For this study, a data set containing 25 paediatric patients, acquired at 4 UK centres was used. The data were gathered from the Children’s Cancer and Leukaemia Group (CCLG) functional imaging of tumours database.^37^ 23 of the data sets were acquired pre-diagnosis, and 2 of the data sets were acquired post-diagnosis. One of the post-diagnosis patients underwent a biopsy and chemotherapy, and the other underwent a surgical resection. The acquisition protocols used are summarised in Table 1. The patient data are from an imaging study entitled “CNS 2004 10 Functional Imaging of Tumours” (NRES REC ref: 04/MRE04/41). This is a multicentre paediatric study with ref: RG_09–028 and ethics ref: ERN_11–1170. Informed parental consent was obtained for all patients included in the study.

**Table 1.** A summary of each of the acquisition protocols used, the number of patients who were recorded using that protocol and the number of signal–time courses used

Centre	No. of patients	No. of time courses	Field strength (T)	Pre-bolus	Sequence	Flip angle (°)	TE (ms)	TR (ms)	Voxel size (mm)
1	3	139	3	Yes	GE-EPI	20	40	1829–4865	2.5 × 2.5 x 3.5
1	5	193	1.5	Yes	GE-EPI	20	40	1490–1643	2.4 × 2.4 x 5
1	3	112	3	No	sPRESTO	7	22	15	3.4 × 3.4 x 3.5
2	5	177	3	Yes	GE-EPI	75	40	1335–2343	1.75 × 1.75 x 4
3	3	166	3	No	GE-EPI	45	29	1570	3.4 × 3.4 x 3.5
4	3	112	3	Yes	GE-EPI	20	40	1865	2.5 × 2.5 x 3.5
4	2	96	1.5	No	sPRESTO	7	25	17	3.4 × 3.4 x 3.5
4	1	16	3	No	sPRESTO	7	24	16	1.8 × 1.8 x 3.5

In the Sequence column, GE-EPI = Gradient Echo – Echo Planar Imaging, and sPRESTO = Sensitivity Encoded (SENSE) Principles of Echo-Shifting with a Train of Observations.^38^

**Figure 1.**
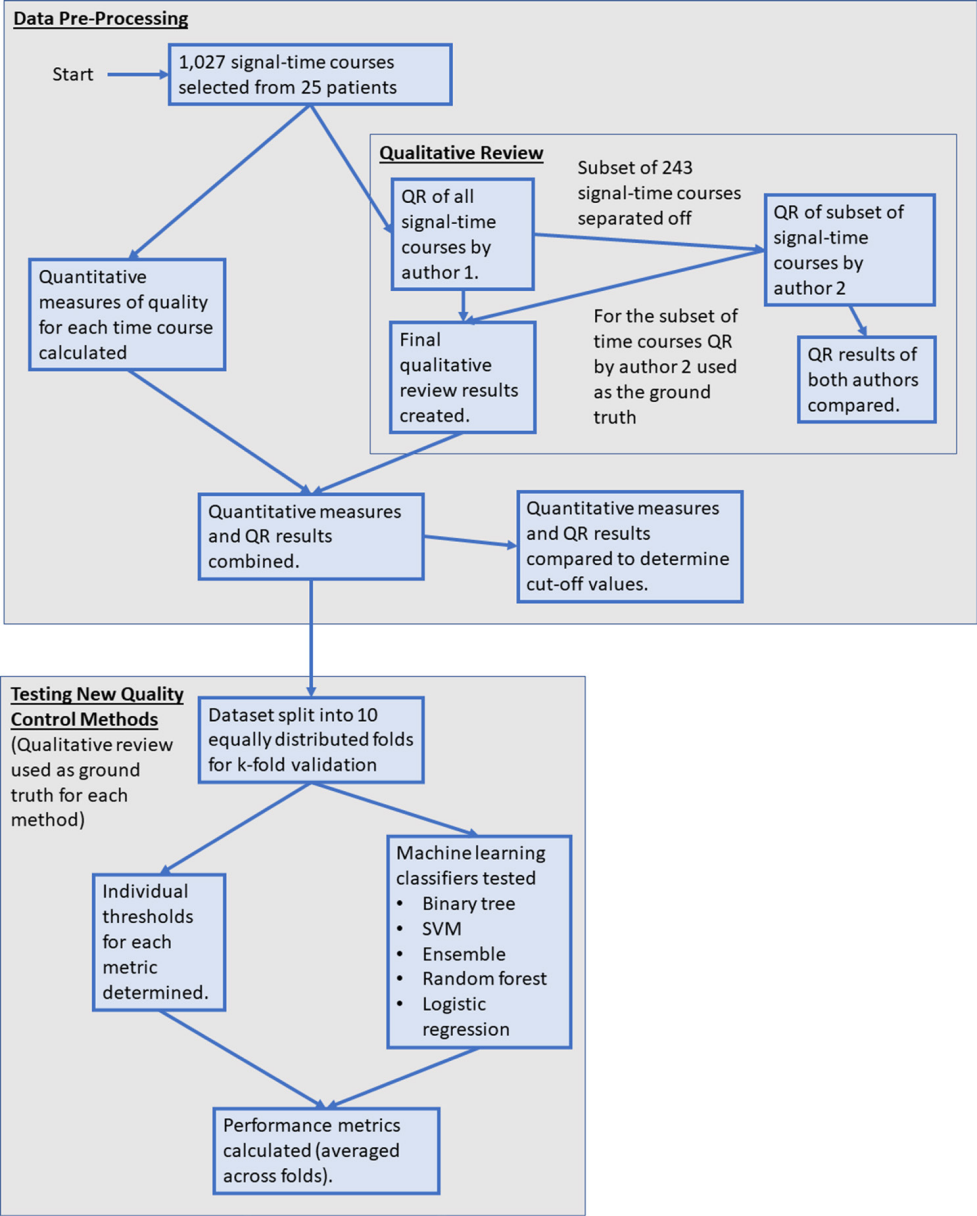
A flowchart summarising the various stages of the work presented within this paper. QR, qualitative review; SVM, support vector machine.

Figure 1 summarises the processes involved in developing an automated QC aid. Details of each step are given below.

#### QR of patient data

QR of 1027 signal–time courses, extracted from 25 patients, was performed. A large number of patients were used to ensure a range of acquisition protocols and artefacts were included. Artefacts were those observed in normal clinical practice when scanning patients. Table 1 summarises how many signal–time courses came from each acquisition protocol. Signal–time courses were randomly selected from pre-defined regions within each patient, which included: grey matter (GM), white matter (WM), the edge of the brain, the edge of the ventricles and the cerebellum. All signal–time courses were selected from slices which did not contain any tumour, by selecting supratentorial signal–time courses from patients with infratentorial tumours and infratentorial signal–time courses from patients with supratentorial tumours. Tumour diagnosis information was obtained from the CCLG database. All signal–time courses were assessed using QR by Author 1 (PhD student with 3 years’ experience), and a randomly selected subset of 243 signal–time courses were additionally assessed by Author 2 (Clinical scientist with 8 years’ experience). QR to assess data quality was carried out using the guidance from the ASFNR recommendations.^11^ This involved assessing whether a clear signal drop was present and the level of noise within the baseline and the rest of the signal. Signal–time courses were then given a score of 1 (accepted) or 0 (rejected) based on this assessment. For the subset of signal–time courses reviewed by two reviewers, the scores from Author 2 were considered to be the ground truth and were used for the determination of thresholds and in the training of ML classifiers.

The percentage disagreement between the two reviewers and the Cohen’s κ for interrater reliability were calculated for the subset of 243 signal–time courses assessed by both reviewers. All statistical analysis was carried out in R (R Foundation for Statistical Computing, Vienna, Austria, v. 3.5.0).

#### Calculating the quantitative measures of quality

Signal drop-to-noise ratio (SDNR), root mean square error (RMSE), full width half maximum (FWHM) and percentage signal recovery (PSR) were used as quantitative measures of signal–time course quality. These were calculated for each of the 1027 signal–time courses which had previously undergone QR. SDNR was calculated using equation 1, with the signal drop defined as the difference between the mean baseline and mean of the first pass minima and the two adjacent dynamics. This is a similar measure to the contrast-to-noise ratio applied in work by Digernes et al, except it is calculated from the signal–time course instead of the relaxation rate curve.^39^

(1) $$\begin{array}{c} SDNR=\frac{SignalDrop}{StandardDeviation\;in\;Baseline} \end{array}$$

RMSE was calculated by fitting a version of the simplified γ variate function,^40^ shown in equation 2, to the first pass of the signal–time course.

(2) $$\begin{array}{c} y\left(t\right)=c-Kt^{\alpha }e^{\frac{-t}{\beta }} \end{array}$$

Where y(t) is the fit, t is the time, c is the average baseline signal, and α, β and K are shape coefficients. The RMSE value from this fit was normalised to the area of the first pass.

The FWHM was calculated as the width of the first pass (in seconds) at half the signal drop.

The PSR was calculated from equation 3, with T_2_^*^ recovery defined as the difference between the mean post-bolus signal and the mean of the first pass minima and the two adjacent dynamics.^11^

(3) $$\begin{array}{c} PSR=\frac{T_{2}^{*}Recovery}{SignalDrop}\times 100 \end{array}$$

**Figure 2.**
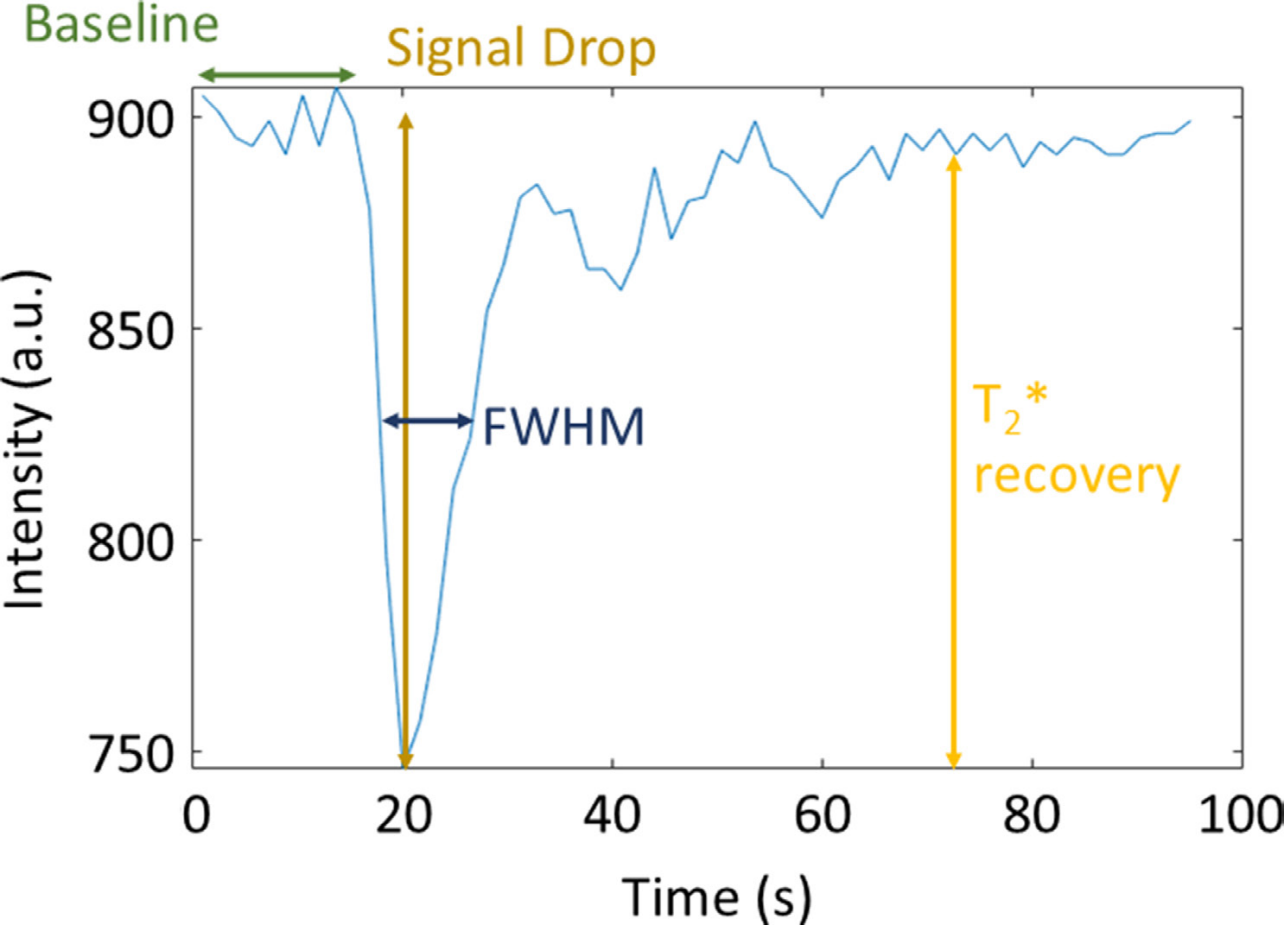
Example signal–time course showing which features of the signal–time course are used to calculate the quantitative measures. FWHM, full width half maximum.

To calculate these measures, it was necessary to define the dynamics where the baseline ended, and the post-bolus started. The baseline end was determined by calculating the moving mean (with sliding window of three) and cumulative mean of the signal–time course, starting from the first dynamic, and finding the dynamic where the means diverged. The start of the post-bolus was determined using the same process but starting from the last timepoint. The first pass was defined as the region between the end of the baseline and the start of the post-bolus. Figure 2 shows an example signal–time course and the features used to calculate the quantitative measures.

#### Thresholds from QR

Quality thresholds for SDNR, RMSE, FWHM, and PSR, were determined using the QR results from the 1027 signal–time courses that underwent QR (Figure 1). Thresholds were determined using k-fold cross-validation (CV), with *k* = 10. Data are separated into k equally sized folds, with (k-1) folds used as training data, and the remaining fold used as testing data, from which the performance metrics are calculated. This process is repeated until all folds have been used as testing data.^41^ The separation of signal–time courses into folds was stratified to ensure an even distribution of accepted and rejected signal–time courses in each fold. The centre or data set the signal–time courses came from was not considered when separating into folds.

For each fold, threshold values were determined from the training data. Sensitivity, specificity, precision, classification error and area under curve (AUC) from a receiver operator curve (ROC) were calculated as performance metrics, by applying the thresholds to the testing data. Mean thresholds and performance metrics were calculated by averaging across the folds.

SDNR and RMSE quality thresholds were determined using sensitivity *vs* specificity plots. For each fold, the SDNR threshold was varied over each SDNR value within the training data, and the sensitivity and specificity were calculated from applying the threshold to the training data and comparing to the QR results. The optimal threshold was the value where sensitivity equalled specificity. This process was repeated for the RMSE values.

Upper and lower thresholds of quality were determined for FWHM and PSR, respectively. For each fold, the parameter values from the training data were ordered in ascending value. The signal–time courses with the smallest and largest FWHM or PSR values, respectively, that passed QR were identified. These values were used as thresholds, with any signal–time course with an FWHM or PSR between the two thresholds classed as good quality.

#### Combining quantitative measures using ML

ML classification was carried out using the ML toolbox in Matlab (The MathWorks, MA, 2019a).^42^ Classification was carried out using the data set of 1027 signal–time courses used for determining thresholds from QR. SDNR, RMSE, FWHM and PSR values were used as predictors for classifier training and the QR scores (1 = passed QR, 0 = failed QR) used as the target outputs. Hyperparameter optimisation was applied for each classifier and k-fold CV with *k* = 10 was used. As previously the k-fold validation was stratified to ensure that there an even distribution of accepted and rejected signal–time courses in each fold, but the centre or data set the signal–time course came from was not taken into consideration, as the aim of this work is to apply the final classifier to a wide range of patient data. The classifiers used were binary tree, support vector machine (SVM), ensemble, random forest, and logistic regression. These classifiers were selected to ensure that a wide range of classification methods were applied to the data. This will help to ensure that the optimal ML classifier is chosen. The average sensitivity, specificity, precision, classification error and AUC were calculated for each classifier. Further details on the ML, including the hyperparameter optimisation and the results of the hyperparameter optimisation, can be found in the appendix.

#### Application to patient data

Each of the thresholds of the quantitative measures of quality and the best performing ML classifier were applied to signal–time courses obtained from one slice of patient data acquired using the acquisition protocol described in row 3 of Table 1. A quality map was created for each method, showing which voxels had passed QC and which had failed.

## Results

### QR of patient data shows good agreement between reviewers, and that there is a region of uncertainty where it is difficult to classify signal–time courses

Table 2 splits the signal–time courses assessed by two reviewers into three groups (all signal–time courses, all 1.5 T signal–time courses, and all 3 T signal–time courses) and summarises the percentage disagreements and Cohen’s κ for each group. Across the entire subset, the signal–time courses where there were disagreements between reviewers had median SDNR of 5.4 (range 3.3–56.4), median RMSE of 0.020 (range 0.005–0.058), median PSR of 84.5% (range 52.5–107.1%), and median FWHM of 7 s (range 4- 15 s).

**Table 2.** A summary of the differences between reviewers, in terms of percentage difference and Cohen’s κ, across all time courses, all time courses recorded at 1.5 T, and all time courses recorded at 3 T

Group	No. of time courses	Percentage difference (%)	Cohen’s κ
All signal–time courses	243	6.58	0.84
1.5 T signal–time courses	81	12.35	0.73
3 T signal–time courses	162	3.70	0.79

**Figure 3.**
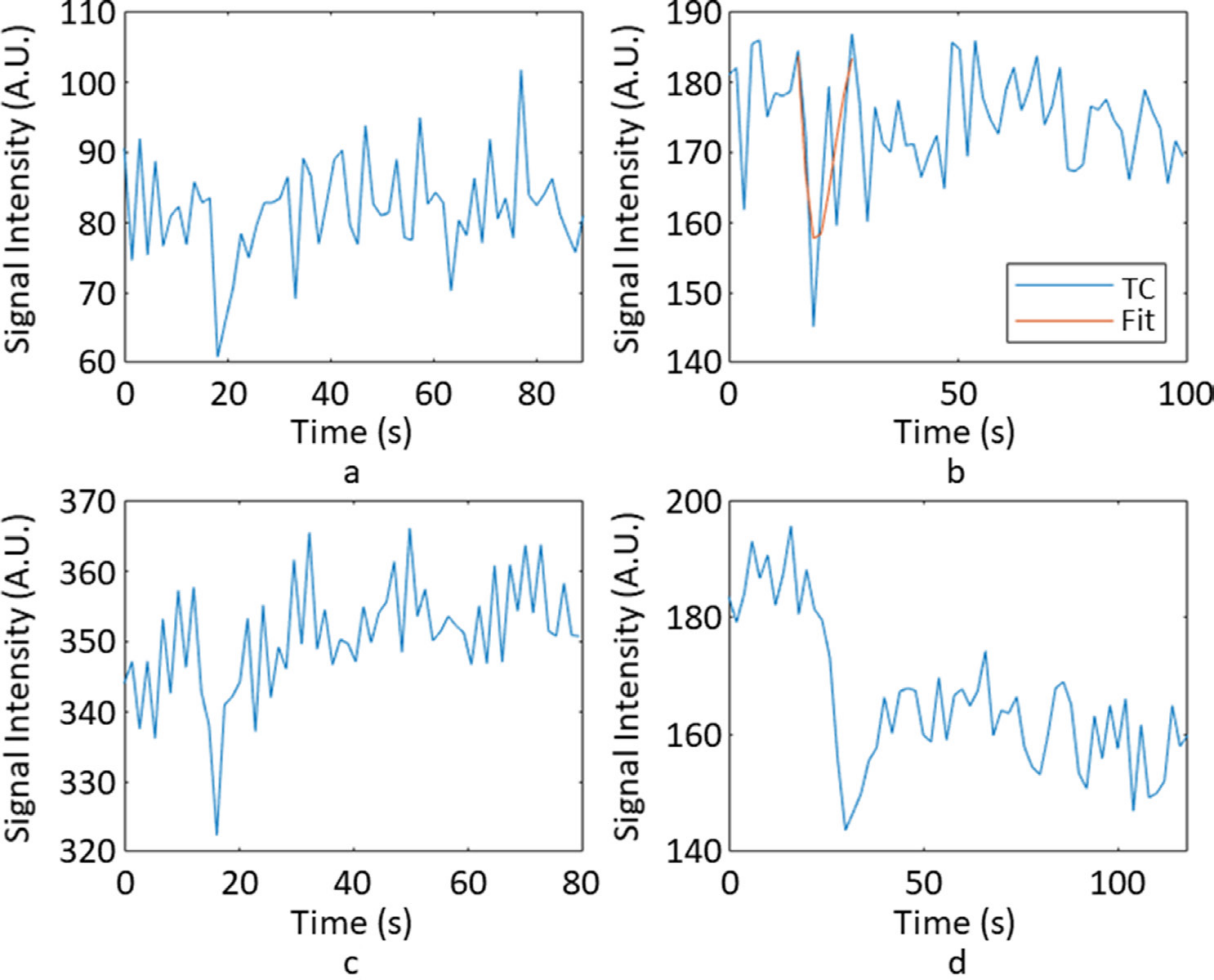
An example of signal–time course that failed QR for differing reasons. (a) has a low SDNR value of 2.7, (b) has a large RMSE value of 0.129, (c) has a small FWHM value of 1.8s (d) has a low PSR value of 36.9%. FWHM, full width half maximum; PSR, percentage signal recovery; RMSE, root mean square error; SDNR, signal drop-to-noise ratio.

Figure 3 shows some examples of signal–time courses that failed QR for different reasons: 3(a) failed due to a small signal drop in comparison to the noise in the baseline, leading to a low SDNR; 3(b) failed due to a noisy first pass, leading to a large RMSE value; 3(c) failed due to a very narrow first pass, which lead to a very small FWHM value; 3(d) failed due to a low T_2_^*^ recovery, resulting in a low PSR value. Below an SDNR value of 2.8, no signal–time courses passed QR, whilst above SDNR of 56.4, no signal–time courses failed QR. Above an RMSE value of 0.0846, no signal–time courses passed QR, whilst below an RMSE value of 0.0055, no signal–time courses failed QR. No signal–time courses with FWHM values less than 3 s or greater than 19 s passed QR. No signal–time courses with PSR values less than 43% or greater than 131% passed QR.

### SDNR is the most important quantitative measure, but other measures are needed

**Figure 4.**
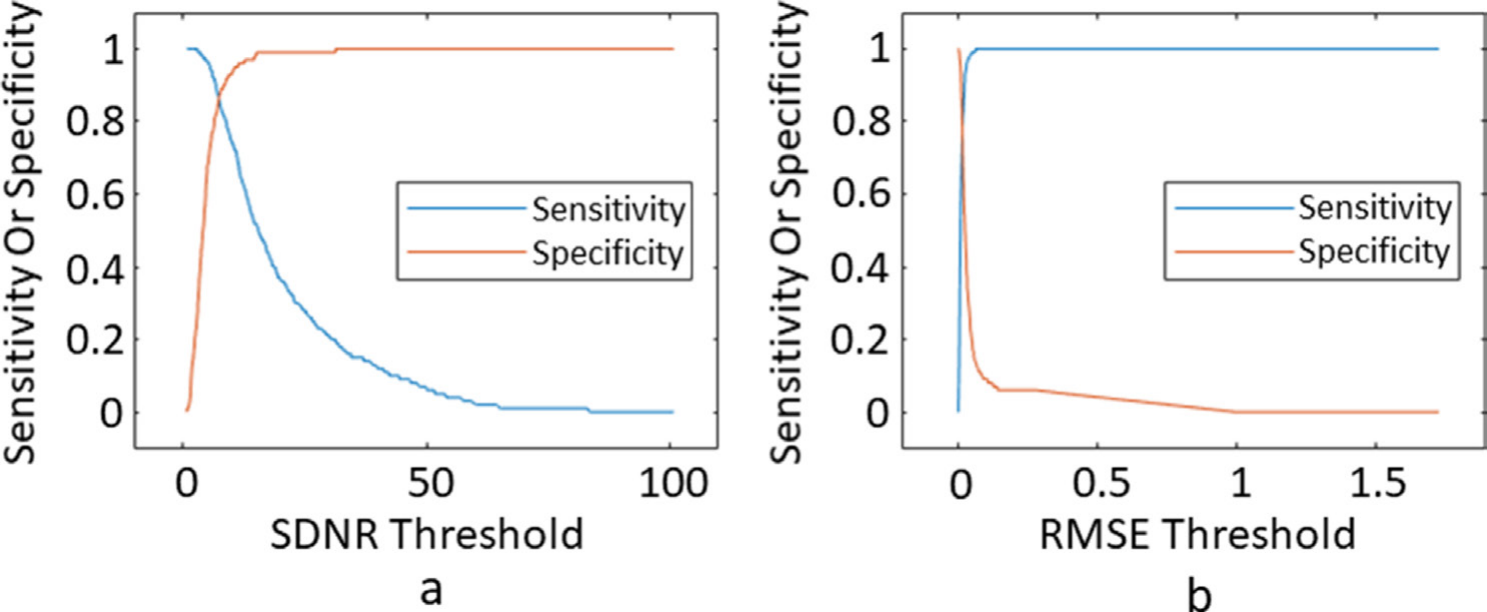
Sensitivity vs.specificity plots for (a) SDNR, (b) RMSE. RMSE, root mean square error; SDNR, signal drop-to-noise ratio.

**Figure 5.**
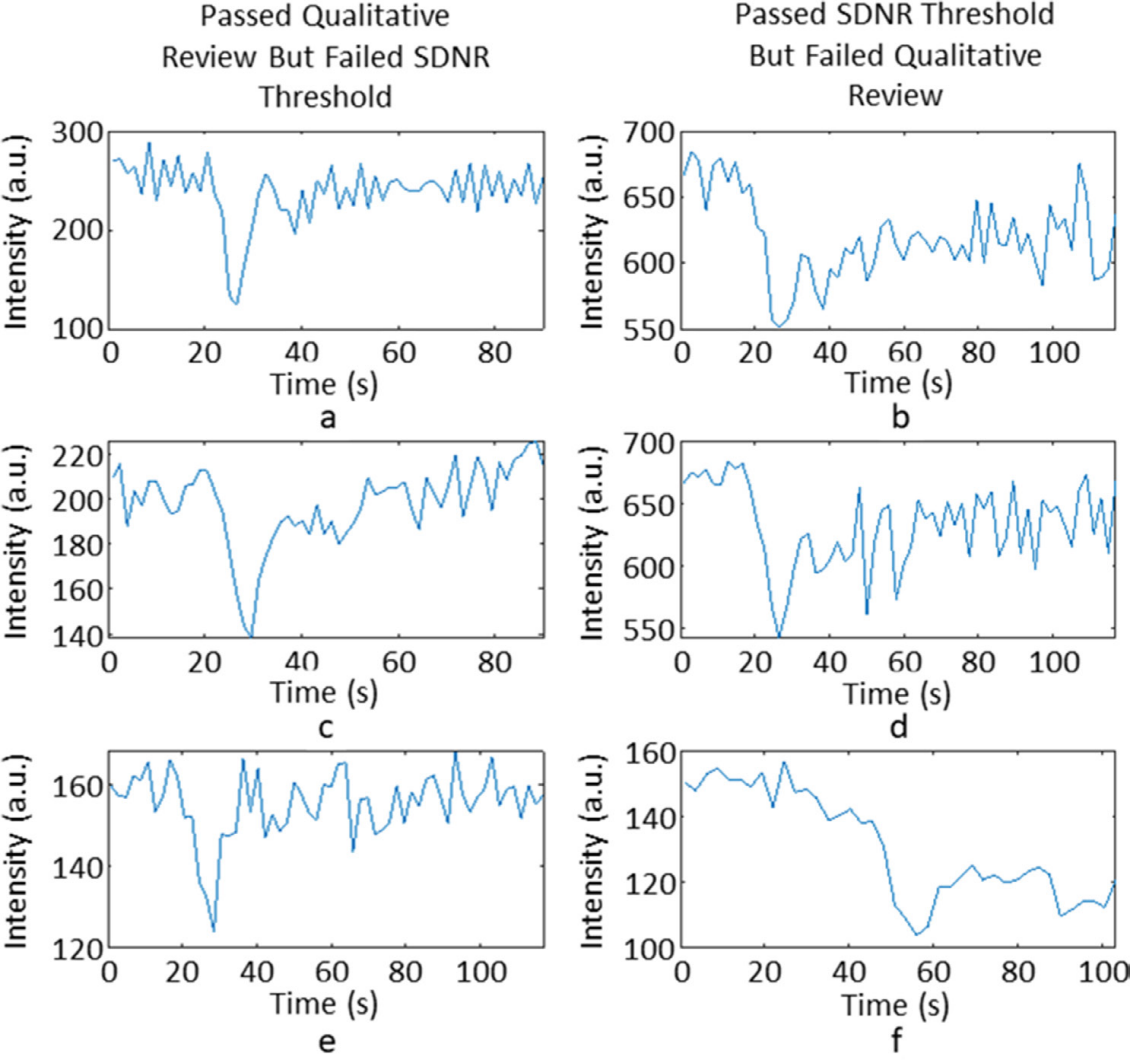
A demonstration of the disagreements between the QR results and the SDNR threshold. The left column (a, c, e) shows signal–time courses that passed QR but failed the SDNR threshold. The right column (b, d, f) shows signal–time courses that passed the SDNR threshold but failed QR. QR, qualitative review; SDNR, signal drop-to-noise ratio.

Figure 4 shows example sensitivity *vs* specificity plots for one of the folds. Averaging across all folds produced an SDNR threshold of 7.6, an RMSE threshold of 0.019, FWHM lower and upper thresholds of 3 s and 19 s, and PSR lower and upper thresholds of 42.9 and 130.4%. The average performance measures are summarised in Table 3. Figure 5 shows example signal–time courses where there were disagreements between the QR results and the SDNR threshold for acceptance of quality. Out of the three signal–time courses that passed the SDNR threshold but failed QR, all three passed the FWHM and PSR thresholds, and one passed the RMSE threshold. All three signal–time courses failed QR because of issues with the post-bolus signal, which was not picked up by SDNR. Out of the three signal–time courses that passed QR but failed the SDNR threshold, one passed the RMSE threshold and two passed the FWHM and PSR thresholds.

**Table 3.** Summary of the average threshold values, sensitivity, specificity, precision, classification errors and AUCs for each of the quantitative measures

Quantitative measure	Threshold(s)	Sensitivity	Specificity	Precision	Classification error (%)	AUC
SDNR	7.6	0.86	0.86	0.93	14.2	0.83
RMSE	0.019	0.79	0.79	0.89	21.8	0.75
FWHM (s)	3, 19	1.00	0.18	0.74	24.8	0.85
PSR (%)	42.9, 130.4	1.00	0.12	0.73	26.4	0.84

AUC, area under the curve; FWHM, full width half maximum; PSR, percentage signal recovery; RMSE, root mean square error; SDNR, signal drop-to-noise ratio.

### Combining quantitative measures to assess data quality reduces classification error and ML classifiers offer an automated method to do this

**Figure 6.**
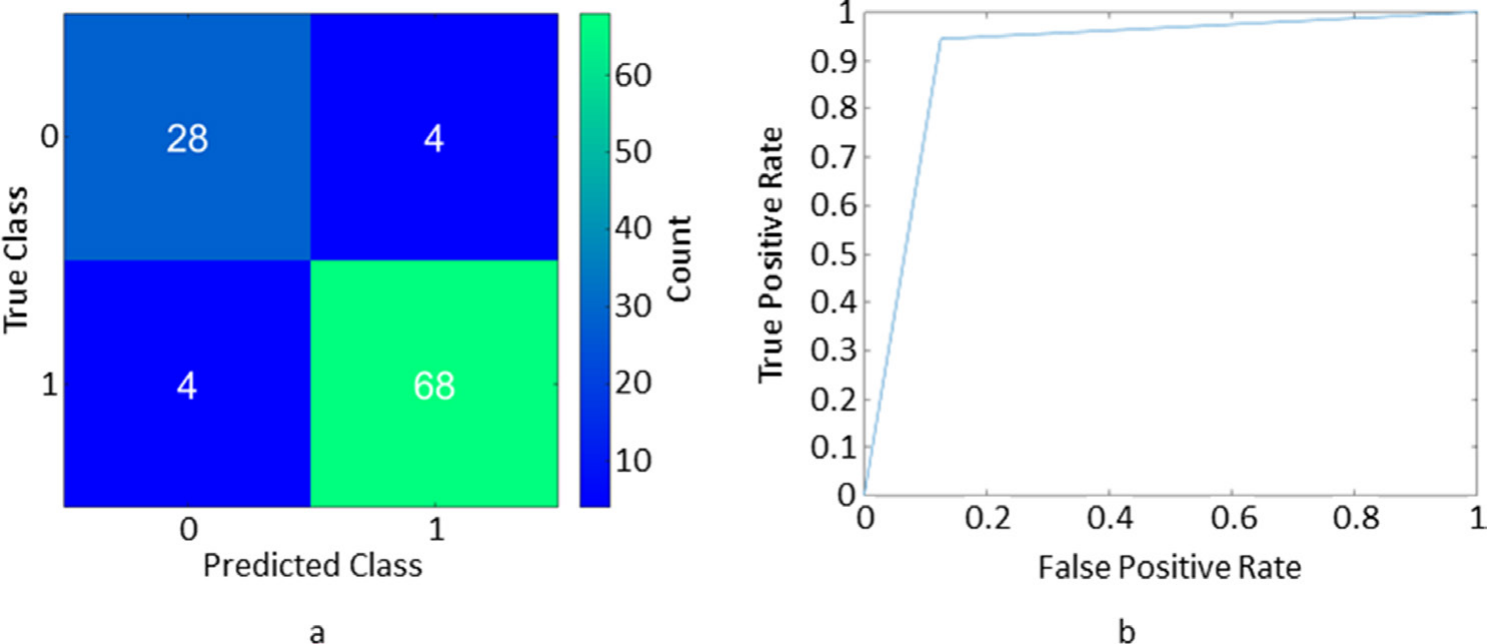
A confusion matrix (a) and an ROC curve (b) from the best performing fold of the best performing classifier (Random forest). The ROC curve gave an AUC value of 0.90. AUC, area under the curve; ROC, receiver operating curve.

**Figure 7.**
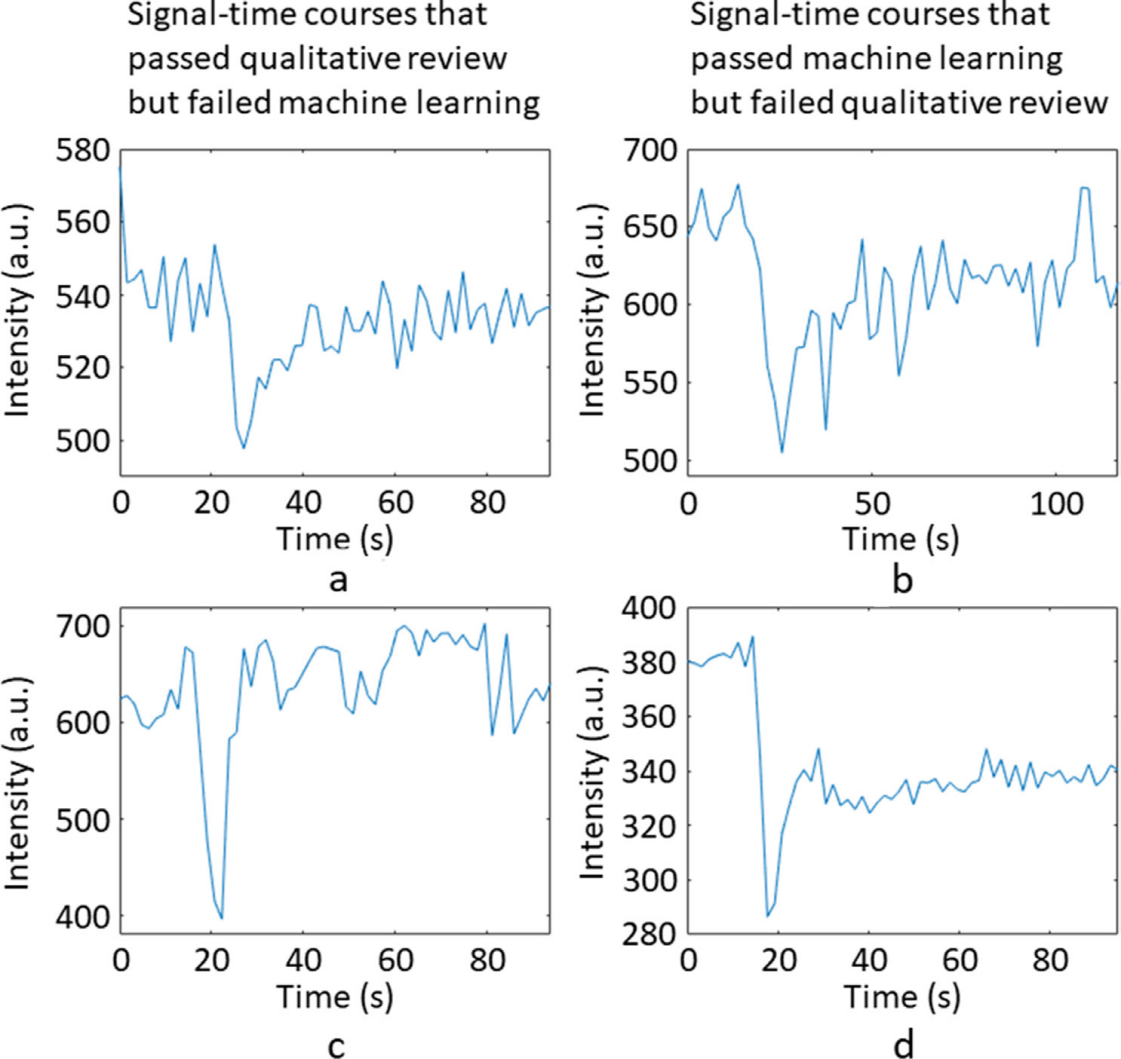
An example of some of the agreements and disagreements between the ML results and QR: (a, c) show signal–time courses that failed ML but passed QR, (b, d) show signal–time courses that passed ML but failed QR. ML, machine learning; QR, qualitative review.

Table 4 summarises the average performance measures for each of the ML classifiers. The classifier with the lowest classification error was the random forest, producing sensitivity, specificity, precision, classification error and AUC of 0.94, 0.83, 0.93, 9.3% and 0.89, respectively. Figure 6 shows an example of the confusion matrix and the ROC curve from the best performing fold of the best performing classifier. Figure 7 shows examples of the disagreements between the QR and the ML results. Details of the hyperparameter optimisation and its results for the best performing classifier can be found in the appendix.

**Table 4.** Summary of the performance metrics for each machine learning classifier

Classifier	Sensitivity	Specificity	Precision	Classification error (%)	AUC
Binary Tree	0.93	0.80	0.92	11.4	0.87
SVM	0.93	0.84	0.93	9.5	0.89
Ensemble^a^	0.94	0.82	0.92	9.5	0.89
Random forest	0.94	0.83	0.93	9.3	0.89
Logistic regression	0.93	0.83	0.92	10.3	0.88

AUC, area under the curve; SVM, support vector machine.

a The Bag method was selected the by the hyperparameter optimisation.

### When applied to one slice of patient data ML passed more signal–time courses than the SDNR threshold

**Figure 8.**
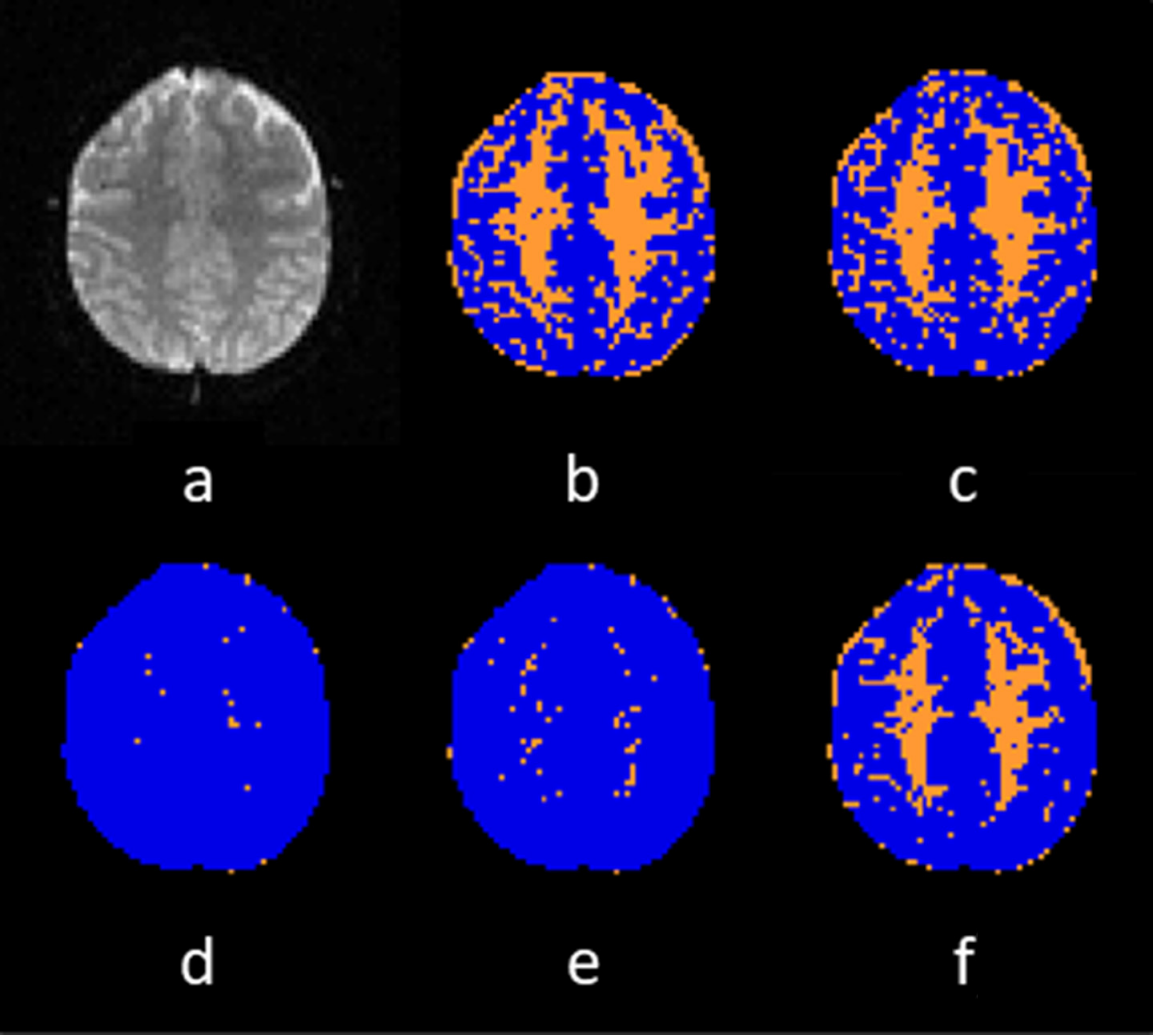
(a) An axial slice from a patient data set recorded at 1.5 T, and the resulting quality maps from applying (b) the SDNR threshold, (c) RMSE threshold, (d) PSR threshold, (e) the FWHM threshold, and (f) the ML classifier, as QC methods. Blue pixels represent time courses that passed QC and orange pixels represent time courses that failed QC. AUC, area under the curve; FWHM, full width half maximum; ML, machine learning; PSR, percentage signal recovery; RMSE, root mean square error; QC, quality control; SDNR, signal drop-to-noise ratio.

Figure 8 shows the quality maps produced by applying the thresholds as obtained from the QR of each of the described metrics and the random forest ML classifier, respectively, to one slice of patient data. Blue pixels represent signal–time courses that passed the respective QC method, whilst orange pixels represent those that failed. Table 5 summarises the percentage of signal–time courses that passed each method.

**Table 5.** % of signal–time courses passed by each quality control method

Quality control method	% of signal–time courses passed
SDNR threshold	60.97%
RMSE threshold	66.18%
PSR threshold	99.12%
FWHM threshold	97.44%
Random forest ML classifier	75.55%

FWHM, full width half maximum; ML, machine learning; PSR, percentage signal recovery; RMSE, root mean square error; SDNR, signal drop-to-noise ratio.

## Discussion

Our study shows that although QR can be used to assess data quality, there are a range of signal–time courses which are difficult to classify. Automated quality control methods using simple metrics can be developed using the results of QR. Combining multiple metrics using ML results in fewer signal–time course misclassifications than using individual metrics. However, selecting a set of metrics to fully describe a signal–time course and all of its potential artefacts is challenging. We applied the automated QC methods to paediatric data in this work, however they are also applicable to adult data.

The signal–time courses assessed by two reviewers show a low discordance between reviewers, due to a low percentage of disagreements and a Cohen’s κ value of 0.83, which shows excellent agreement.^43^ The two reviewers found it harder to agree on whether to pass signal–time curves from data sets acquired at 1.5 T, due to their reduced SDNR. For the entire subset, the ranges of SDNR and RMSE values for signal–time courses where there were disagreements between the reviewers and for signa–-time courses that were within the region of uncertainty are both large. This shows that a signal–time course with a large SDNR is not guaranteed to be good quality, as other factors may also affect quality, including, *e.g.* a distorted first pass.

The automated QC methods presented in this work assess data quality on a voxel-wise basis, which allows for more data to be assessed than in QR. A typical DSC-MRI data set will have tens of thousands of signal–time courses. Assessing all of them by eye is not possible, so QR generally involves assessing a small subset of the whole data set. Our automated QC methods are ML classifiers, trained using signal–time courses from “normal brain”, which could lead to the exclusion of pathology related low perfusion signal–time courses, which may be clinically useful. Large amounts of WM, which is suggested by consensus guidelines as the tissue to use for normalising rCBV values,^19^ may also be excluded as it is less perfused than GM. A better approach may be to use voxel-wise QC to give an overall assessment of data quality, which could then be used to decide whether a data set is of sufficient overall quality to be included in a study. An alternative to this would be to assess the quality of an average signal–time course from a data set. This would be quicker than a voxel-wise analysis but could produce misleading results—an average signal–time course will “average out” noise and regions of artefacts from the data.

The SDNR threshold defined the minimum SDNR for data to be accepted and was the best-performing individual measure, giving the most similar results to QR. This is expected as SDNR defines how visible the signal drop is, which is a key part of assessing data quality by QR.^11^ However, SDNR is reduced in low perfused tissues, such as white matter or some low-grade tumours, and this may lead to the exclusion of some of these signal–time courses, discarding clinically useful information. The RMSE threshold gave poorer performance across all the performance measures compared to SDNR. The FWHM and PSR thresholds give AUC values comparable to the other quantitative measures and a similar classification error to the RMSE threshold. Both resulted in very good sensitivity but poor specificity.

Multiple factors affect DSC-MRI data quality, and a single measure cannot cover them all. Figure 5 illustrates the difficulties of trying to classify signal–time course quality purely on SDNR. Difficulty in defining a single threshold for each quantitative measure shows the need for combined measures to assess data quality. This agrees with work by Akella et al^22^ where multiple quantitative measures including, the failure rate of fitting a gamma-variate to the first pass, mean FWHM, and mean PSR were calculated from the signal–time courses in each data set and used to determine data quality. Cut-off values were calculated using a 99% one-sided confidence interval. Data sets that did not fall within the cut-off values for at least one metric were classed as poor quality.^22^ Our work presented here differs in that thresholds for quality are determined using QR results instead of confidence intervals. Combining measures using ML classifiers, leads to improved classification error compared to individual thresholds as shown in Table 4. The random forest classifier gave the lowest classification error, but offers only a minor improvement in performance measures compared to the other classifiers. Therefore, any of the classifiers tested would be suitable.

The ML classifier offers improved sensitivity, classification error and AUC, compared to the SDNR threshold. There is little change in specificity and precision, suggesting that the main improvement in performance comes from a reduction in the number of false negatives, with little change in the number of false positives. ML also has a similar classification rate to the percentage disagreements in QR between reviewers, which implies that it is as accurate as QR at least for these data sets. Therefore, when the quality control methods are applied to a patient data set, the ML classifier passes a higher percentage of signal–time courses than the SDNR threshold, as shown in Figure 8 and Table 5. The lack of reduction in the number of false positives is likely due to the current quantitative measures not being able to identify all the artefacts that DSC-MRI is susceptible to.

The ML classifiers were trained using k-fold validation. Stratified k-fold validation was used to ensure that there was an even distribution of accepted and rejected signal–time courses in each fold. The centre and data set the signal–time courses originated from was not considered so this was applied on a signal basis rather than a subject basis. This is because the aim of this work is to train a classifier which can be applied to a wide range of patient data, so it needs to be capable of handling data from different centres acquired with different acquisition parameters. Therefore, splitting the data in a subject basis could bias the classifier and reduce its performance.

Currently, the results of qualitative review are applied to a series of quantitative measures which are calculated from the signal–time courses. If a convolutional neural network (CNN) was used in place of the ML classifier, then it could be trained using the signal–time courses directly, rather than calculating measures from the signal–time courses. However, currently there are not enough data to train a CNN type model. This is something that could be investigated in the future once more data have been acquired.

The use of a pre-bolus or single-bolus injection protocol in paediatric data affects the SDNR of the signal–time courses by reducing the signal drop. In adults, a pre-bolus of contrast agent may be given in addition to a full dose of contrast agent, increasing the overall SNR. However, in paediatrics, the European Society for Paediatric Oncology (SIOPE) recommends that paediatric patients should only receive a single-dose of contrast agent, due to concerns over gadolinium deposition.^44^ Splitting a single-dose in order to give a pre-bolus will therefore cause a reduction in SDNR in the DSC-MRI acquisition.

Most DSC-MRI studies use the ASFNR recommendation of QR to assess data quality.^11^ Automated QC using statistical thresholds has previously been presented by Akella et al.^22^ Our method differs as the thresholds and ML classifier are trained on the results of QR. An alternative way to assess data quality is for a radiologist to assess the quality of the perfusion maps produced. This could either mean assessing the diagnostic quality of the perfusion maps,^45,46^ assessing the presence of susceptibility artefacts,^47^ or assessing the visibility of a certain region of the brain.^48^ However, these methods are not automated and risk artefacts being misinterpreted as pathology.

In order to calculate the metrics presented in this study, it is necessary to establish the end of the baseline signal in each signal–time course. There are established methods for determining the end of the baseline, *e.g.* Carroll et al^49^ present a method which uses adaptive thresholds calculated from the standard deviation of the pre-contrast signal, defining a set number of time points from which to calculate the adaptive thresholds.^49^ The method we have presented is better suited to a multicentre data set with variable injection protocols, where the number of dynamics in the baseline may vary between centres.

There are some limitations to this study. Firstly, the patient “training data set does not include every type of artefact, such as susceptibility artefacts or insufficient dynamics to capture the full passage of the contrast agent. There may be cases where the classifier misclassifies a signal–time course with an artefact appearing for the first time. The training data set is also made up of signal–time courses acquired with specific and consistent acquisition protocols. Any changes in acquisition protocol will affect the signal values and therefor the quantitative measures, *e.g.* PSR can vary with acquisition protocol.^13^ So, thresholds would need to be recalculated at different centres.

Secondly, whilst these methods were tested on signal–time courses from slices of brain that did not contain tumour or other definite pathology, they may still not be “normal tissue”. The quantitative measures may differ in diseased tissue or tissue that has been exposed to treatments such as radiotherapy and so the thresholds and methods presented may not be suitable to all circumstances. For example, PSR has been used to exclude signal–time course with unusual post-bolus signals. However, PSR will be affected by contrast agent leakage due to the breakdown of the blood–brain barrier in tumours. In this case, a leakage correction method, such as the Boxerman-Schmainda-Weisskoff method^50^ could be applied prior to quality assessment. In this study, leakage correction was not applied as no tumour signal–time courses were included.

Finally, although the classifier offers an automated QC method, it is still based on QR so still has an element of subjectivity to it.

## Conclusions

QR of individual signal–time courses by two reviewers showed good agreement on the signal–time courses they assessed. ML classifiers trained on QR results offer an automated method to assess the quality of an entire data set. Although SDNR was a good indicator of quality, using only a single measure to determine data quality risks misclassification of signal–time courses. Combining SDNR with RMSE, FWHM and PSR improves classification, and achieves a misclassification rate similar to the discordance rate of QR. We have shown that ML classifiers trained on QR can be used to assess quality of DSC-MRI signal–time courses obtained from normal brain in this paediatric data set.
